# Fisetin Attenuated Oxidative Stress-Induced Cellular Damage in ARPE-19 Human Retinal Pigment Epithelial Cells Through Nrf2-Mediated Activation of Heme Oxygenase-1

**DOI:** 10.3389/fphar.2022.927898

**Published:** 2022-06-16

**Authors:** Cheol Park, Jeong Sook Noh, Youngmi Jung, Sun-Hee Leem, Jin Won Hyun, Young-Chae Chang, Taeg Kyu Kwon, Gi-Young Kim, Hyesook Lee, Yung Hyun Choi

**Affiliations:** ^1^ Division of Basic Sciences, College of Liberal Studies, Dong-Eui University, Busan, South Korea; ^2^ Department of Food Science and Nutrition, Tongmyong University, Busan, South Korea; ^3^ Department of Integrated Biological Science, Pusan National University, Busan, South Korea; ^4^ Department of Biological Sciences, Pusan National University, Busan, South Korea; ^5^ Department of Biomedical Sciences, Dong-A University, Busan, South Korea; ^6^ Department of Health Sciences, Dong-A University, Busan, South Korea; ^7^ Department of Biochemistry, College of Medicine, Jeju National University, Jeju, South Korea; ^8^ Research Institute of Biomedical Engineering and Department of Medicine, Catholic University of Daegu School of Medicine, Daegu, South Korea; ^9^ Department of Immunology, School of Medicine, Keimyung University, Daegu, South Korea; ^10^ Department of Marine Life Science, Jeju National University, Jeju, South Korea; ^11^ Department of Convergence Medicine, Pusan National University School of Medicine, Yangsan, South Korea; ^12^ Anti-Aging Research Center and Core-Facility Center for Tissue Regeneration, Dong-Eui University, Busan, South Korea; ^13^ Department of Biochemistry, Dong-Eui University College of Korean Medicine, Busan, South Korea

**Keywords:** fisetin, human retinal pigment epithelial cells, DNA damage, apoptosis, ROS, Nrf2/HO-1

## Abstract

Fisetin is a kind of bioactive flavonol, widely present in various fruits such as strawberries and apples, and is known to act as a potent free radical scavenger. However, the mechanism of action related to the antioxidant activity of this compound in human retinal pigment epithelial (RPE) cells is not precisely known. In this study, we aimed to investigate whether fisetin could attenuate oxidative stress-induced cytotoxicity on human RPE ARPE-19 cells. To mimic oxidative stress, ARPE-19 cells were treated with hydrogen peroxide (H_2_O_2_), and fisetin significantly inhibited H_2_O_2_-induced loss of cell viability and increase of intracellular reactive oxygen species (ROS) production. Fisetin also markedly attenuated DNA damage and apoptosis in H_2_O_2_-treated ARPE-19 cells. Moreover, mitochondrial dysfunction in H_2_O_2_-treated cells was alleviated in the presence of fisetin as indicated by preservation of mitochondrial membrane potential, increase of Bcl-2/Bax expression ratio, and suppression of cytochrome *c* release into the cytoplasm. In addition, fisetin enhanced phosphorylation and nuclear translocation of nuclear factor erythroid 2 related factor 2 (Nrf2), which was associated with increased expression and activity of heme oxygenase-1 (HO-1). However, the HO-1 inhibitor, zinc protoporphyrin, significantly reversed the protective effect of fisetin against H_2_O_2_-mediated ARPE-19 cell injury. Therefore, our results suggest that Nrf2-mediated activation of antioxidant enzyme HO-1 may play an important role in the ROS scavenging activity of fisetin in RPE cells, contributing to the amelioration of oxidative stress-induced ocular disorders.

## Introduction

Oxidative stress-induced retinal disorders are thought to play an important role in the pathogenesis of irreversible vision loss-related diseases, including retinitis pigmentosa, diabetic retinopathy and age-related retinal degeneration (AMD) (Chichagova et al., 2018; Léveillard et al., 2019). The retina is composed of a mono-layer of retinal pigment epithelial (RPE) cells and a variety of neurons, including photoreceptors, and RPE cells provide multiple physiological defenses to maintain photoreceptor homeostasis for vision formation (Yonekawa et al., 2015; Sadda et al., 2020). However, RPE cells are exposed to an environment rich in the production of endogenous reactive oxygen species (ROS) due to excessive oxygen consumption for high metabolic rate of photoreceptors and chronic light exposure (Wang et al., 2021; Eells, 201911). Under normal conditions, these oxidative stress stimuli can be defended by the oxidant scavenging system of RPE cells, but the accumulation of oxidative damage and aging-related decline in antioxidant capacity ultimately contribute to RPE dysfunction (Eells, 201911; Blasiak et al., 2014; Toma et al., 2021). Mitochondria are the main intracellular organelles involved in ROS generation in all types of cells, including RPE cells, and excessive ROS not only causes oxidative damage to DNA, but also destroys mitochondrial membrane integrity, causing mitochondrial dysfunction and apoptotic cell death (Blasiak et al., 2014; Brook et al., 2019; Wang et al., 2021). In this context, protecting RPE cells from oxidative stress could be an effective strategy to prevent or attenuate retina-mediated ophthalmic diseases. Heme oxygenase-1 (HO-1) is a responsive enzyme that catabolizes free heme into carbon monoxide, and the degradation of HO-1 into heme consumes 3 molecules of oxygen, indirectly triggering an inhibitory effect on oxidative damage (Lenzi et al., 2005; Hong et al., 2021a). Accumulated evidences suggested that HO-1 can be activated by various forms of stimuli to protect cells including RPE (Zhou et al., 2020a; Hong et al., 2021a). There has been reported that activation of nuclear factor erythroid-2-related factor 2 (Nrf2)/HO-1 attenuated apoptosis in APRE-19 human RPE cell line (Zhou et al., 2020a; Hong et al., 2021a).

In the past decades, much attention has been paid to bioactive compounds derived from plants as novel candidates for the treatment of oxidative stress-related diseases (Li et al., 2014; Piccolella et al., 2019). Among them, natural products belonging to the flavonol family are known as phytochemicals that have high antioxidant activity but are not toxic, and are widely present in various plants (Kicinska and Jarmuszkiewicz, 2020; Safe et al., 2021). Fisetin (3,3′,4′,7-tetrahydroxyflavone), one of the naturally-occurring flavonols, is abundantly found present in many fruits and vegetables, including apple, persimmon, strawberry, onion, and cucumber, etc., (Khan et al., 2013; Grynkiewicz and Demchuk, 2019). Accumulated data from cell culture and animal models related to human diseases have revealed that fisetin has several beneficial effects, including prevention or alleviation of several chronic diseases, including diabetes, obesity, cardiovascular, and respiratory diseases (Pal et al., 2016; Martel et al., 2020; Sok Yen et al., 2021; Vishwas et al., 2022). Many studies have demonstrated that fisetin has multiple pharmacological activities such as neuroprotective, anti-inflammatory, chemopreventive, anti-angiogenic, anti-tumorigenic, anti-cancer activities without showing any appreciable toxicity (Zhu et al., 2017; Mehta et al., 2018; Liang et al., 2020; Maher, 2021; Ravula et al., 2021). More interestingly, although excessive accumulation of ROS may be a major factor in fisetin-induced apoptosis of cancer cells (Su et al., 2017; Tsai et al., 2019; Imran et al., 2021), the protective effects of fisetin against cell damage to various stimuli are associated with potent antioxidant activity by acting as a potent ROS scavenger (Zhao et al., 2019; Rodius et al., 2020; Molagoda et al., 2021; Ding et al., 2022). Moreover, several previous studies have demonstrated that this compound can modulate the Nrf2/antioxidant response elements (AREs) pathway to exert antioxidant activity (Sim et al., 2020; Jiang et al., 2021; Li et al., 2022). Although Hanneken et al. (Hanneken et al., 2006) have suggested that the antioxidant activity of fisetin in RPE cells could be related to an increased expression of Nrf2 and HO-1, one of the representative downstream factors of Nrf2, the role of this pathway in the antioxidant activity of fisetin in RPE cells is still unclear. Therefore, the present study aimed to investigate the protective effect of fisetin against oxidative damage mimicked by hydrogen peroxide (H_2_O_2_) treatment in human RPE ARPE-19 cells and to explore the underlying mechanisms involved in Nrf2 signaling.

## Materials and Methds

### Cell Culture and Treatment

ARPE-19 cells (CRL-2302™, American Type Culture Collection, Manassas, VA, United States) were used at passages 25 to 29 and grown in Dulbecco’s modified Eagle’s Medium/F-12 added with 10% fetal bovine serum, 100 U/ml penicillin, and 100 U/ml streptomycin at 37°C under atmospheric conditions at 5% CO_2_. All materials necessary for cell culture were provided by WELGENE Inc. (Gyeongsan, Republic of Korea). Fisetin, zinc protoporphyrin (ZnPP) and H_2_O_2_ were obtained from Sigma-Aldrich Chemical Co. (St. Louis, MO, United States). To prepare their stock solutions, they were dissolved in dimethyl sulfoxide (DMSO, Thermo Fisher Scientific, Inc., Waltham, MA, United States) and further diluted in cell culture medium. The cells were pretreated with or without the indicated concentrations of fisetin and/or 5 µM ZnPP for 1 h before inducing oxidative damage with H_2_O_2_ for 24 h.

### Cell Viability Assay

As reported (Gao et al., 2021), Cytotoxicity was assessed using the cell count kit 8 (CCK8) assay kit (Abcam Inc., Cambridge, United Kingdom) to evaluate cytotoxicity according to the manufacturer’s instructions. After treatment with H_2_O_2_ for 24 h in the presence or absence of fisetin and/or ZnPP, CCK8 solution (10 μl/well) was added, and cells were further incubated for 3 h at 37°C. The absorbance was then measured at 480 nm with a microplate reader (Molecular Device Co., Sunnyvale, CA, United States) and normalized to the value of the untreated control group. Each experiment was performed in triplicate.

### Reactive Oxygen Species Measurement

ROS levels were determined using a fluorescent probe, 2,7-dichlorofluorescin diacetate (DCF-DA, Thermo Fisher Scientific, Inc.), according to the manufacturer’s instructions. Briefly, cells were treated with or without fisetin and/or ZnPP for 1 h and then additionally treated with H_2_O_2_ for 1 h. After washing cells with phosphate buffer saline (PBS), cells were preloaded with 10 μM DCF-DA for 30 min at 37°C in the dark. Afterward, the fluorescence intensity was determined by flow cytometry (BD Biosciences, San Diego, CA, United States) as previously described (Noh et al., 2020). In parallel, DCF-DA-stained Images of cells stained with DCF-DA were obtained using a fluorescence microscope (Carl Zeiss, Oberkochen, Germany) as previously described (Na et al., 2020).

### Western Blot Analysis

Whole cell proteins were extracted from cells exposed to H_2_O_2_ for 24 h after incubation for 1 h with or without fisetin and/or ZnPP using a radioimmunoprecipitation assay buffer (Thermo Fisher Scientific, Inc.). In examining cytochrome *c* expression, cytoplasmic and mitochondrial proteins were separated using a Mitochondria/Cytosol Fractionation Kit (Abcam, Inc., Cambridge, MA, United States) according to the protocol. Equal amounts of protein from each lysate were separated by sodium-dodecyl sulfate polyacrylamide gel electrophoresis and transferred to polyvinylidene fluoride membranes (Millipore Co., Burlington, MA, United States). After blocking the membrane with 5% skim milk in Tris-buffered saline containing 0.1% Tween 20, the membranes were probed corresponding primary antibodies (Cell Signaling Technology, Beverly, MA, United States and Santa Cruz Biotechnology, Inc., Santa Cruz, CA, United States) and then allowed to react with horseradish peroxidase-conjugated secondary antibodies (Santa Cruz Biotechnology, Inc.) as previously described (Gao et al., 2021). After that, protein bands were visualized with an enhanced chemiluminescence kit (Bio-Rad Laboratories, Inc., Hercules, CA, United States) and then imaged on a Fusion FX Image system (Vilber Lourmat, Torcy, France). Equivalent loading was confirmed using actin for total proteins and cytochrome *c* oxidase (COX IV) for mitochondrial proteins.

### Comet Assay

Comet assay to investigate the effect of fisetin on H_2_O_2_-induced DNA damage were performed according to the manufacturer’s instructions for the comet assay kit (Trevigen, Inc., Gaithersburg, MD, United States). Briefly, after applied treatment, cells were embedded into melting agarose and fixed onto comet assay slides as previously described (Volobaev et al., 2020). Slides were immersed in lysis buffer at 4°C to solidify and then placed in pre-chilled alkaline buffer provided by the kit. After alkali lysis, the slides with cells were electrophoresed in a horizontal electrophoresis apparatus and the nuclei and comet tails were subsequently stained with ethidium bromide (EtBr) in the dark. Finally, EtBr-stained images were captured under a fluorescence microscope (Carl Zeiss).

### Observation of Nuclear Morphology

To confirm the induction of apoptosis according to the nuclear morphology change, cells exposed to H_2_O_2_ with or without fisetin were fixed with 4% paraformaldehyde (Sigma-Aldrich Chemical Co.) and permeabilized with 0.5% Triton X-100 (Sigma-Aldrich Chemical Co.) as previously reported (Park et al., 2020). After rinsing the cells with PBS, they were stained with 10 μg/ml 4′,6-diamidine-2′-phenylindole dihydrochloride (DAPI) solution (Thermo Fisher Scientific, Inc.) at room temperature for 10 min in the dark and fluorescence micrographs of the nuclei were then monitored using a fluorescence microscope.

### Flow Cytometry for Apoptosis

After being exposed to H_2_O_2_ with or without fisetin for 24 h, apoptosis rates were determined by flow cytometry using an Annexin V fluorescein isothiocyanate (FITC)/propidium iodide (PI) apoptosis detection kit (BD Biosciences, San Diego, CA, United States) according to the manufacturer’s protocol. Afterward, the ratio of the number of FITC-positive cells to the total number of cells was calculated to express the ratio of apoptotic cells through flow cytometry (Liu et al., 2021).

### Caspase-3 Activity Assay

The caspase-3 activity assay was conducted as per manufacturer instructions for the caspase-3 fluorometric assay kit (R&D Systems, Inc., Minneapolis, MN, United States). After treatment, the harvested cells were lysed with lysis buffer prior to addition of N-acetyl-Asp-Glu-Val-Asp p-nitroanilide (caspase-3 substrate) according to the manufacturers’ instructions. After reacting at 37°C for 1 h, the concentration of p-nitroanilide released from the substrate by activated caspase-3 was analyzed using a microplate reader at the absorbance value of 405 nm. The results were expressed as relative fluorescence units relative to control as described previously (Kim et al., 2021a).

### Immunofluorescence Assay for p-Nrf2

After the indicated treatment, the cells were fixed with 4% paraformaldehyde, permeabilized in 0.5% Triton X-100 and then blocked with 2% bovine serum albumin (Sigma-Aldrich Chemical Co.) as described previously (Lee et al., 2021). Subsequently, the cells were probed with anti-p-Nrf2 antibody (Ser 139) at 4°C overnight and then washes with ice-cold PBS. Thereafter, cells were incubated with Alexa Fluor^®^ 594-conjugated secondary antibody and nuclei were counterstained with 10 μg/ml DAPI. After washing cells with PBS, images were analyzed under a fluorescence microscope.

### Heme Oxygenase-1 Activity Assay

The HO-1 activity of cells treated with H_2_O_2_ alone or with fisetin was evaluated by measuring the amount of bilirubin according to the instructions of the HO-1 activity assay kit (Abcam, Inc.). Briefly, the cell lysates were mixed with reaction buffer supplied by the manufacturer and incubated at 37°C for 1 h in the dark with shaking. After stopping the reaction of the sample at 4°C, the amount of bilirubin produced was measured from the difference in optical density obtained at 460 and 530 nm using a microplate reader. HO-1 activity was presented as picomoles of bilirubin/mg protein as described in a previous study (Jo et al., 2021).

### Statistical Analysis

Statistical analysis was performed using the GraphPad Prism V5.0 software (GraphPad Software Inc., La Jolla, CA, United States). All data were reported as mean ± standard deviation (SD). the statistical analyses that were conducted using analysis of variance (ANOVA) and Tukey’s post-hoc test to examine between-group differences, and *p* values <0.05 were considered to indicate statistically significant results.

## Results

### Fisetin Protected ARPE-19 Cells From H_2_O_2_-Induced Cytotoxicity

**FIGURE 1:**
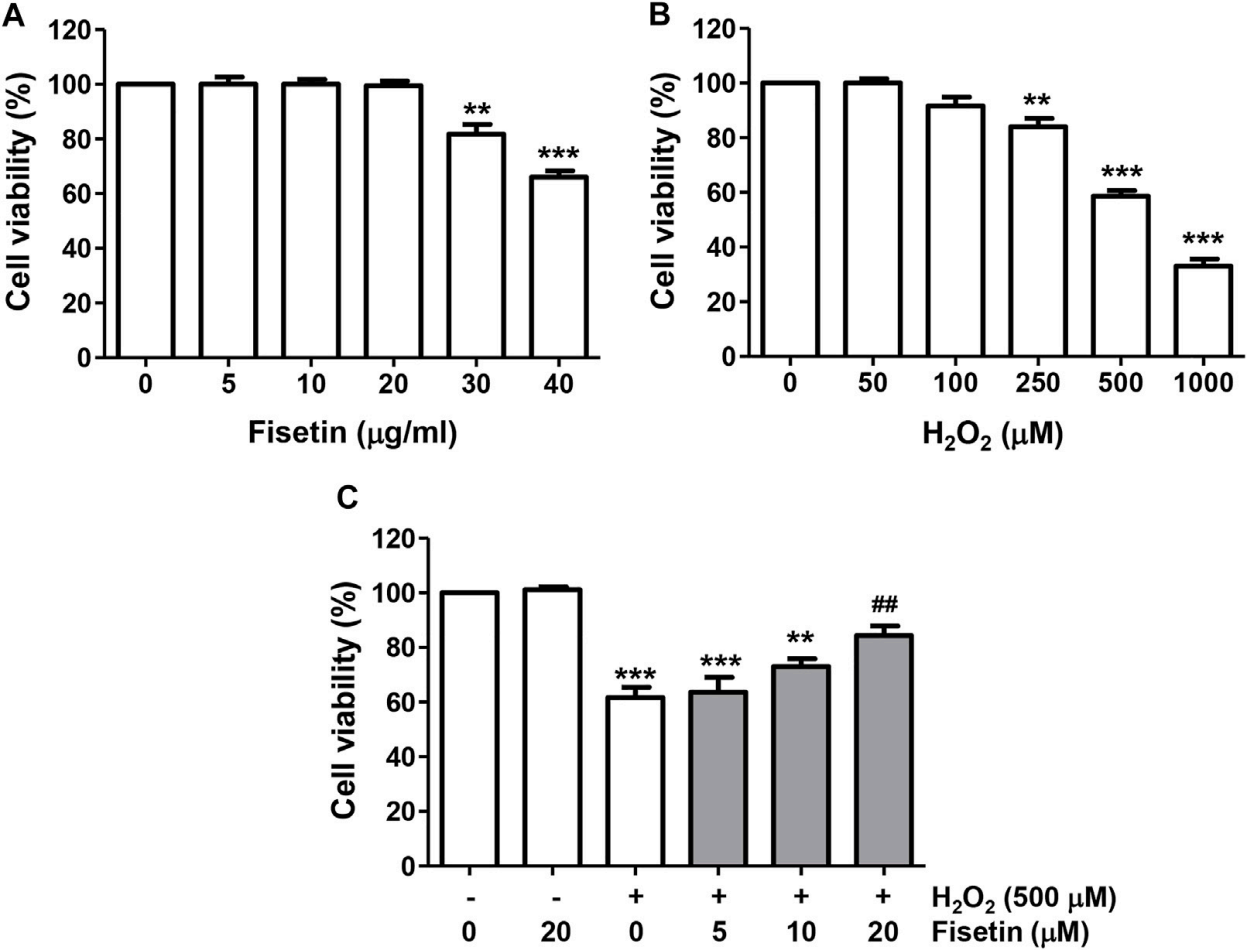
Fisetin prevented reduction of cell viability by H_2_O_2_ treatment in ARPE-19 cells. Cells were treated with different concentrations of fisetin **(A)** or H_2_O_2_ **(B)** for 24 h, or fisetin was pretreated for 1 h and then treated with H_2_O_2_ for an additional 24 h **(C)**, followed by the CCK8 assay. Fisetin have no cytotoxicity up to 20 μM, and H_2_O_2_ result in reduction in cell viability in a dose-dependently. The reduction in cell viability by H_2_O_2_ gradually improved with increasing fisetin pretreatment concentration. Data are graphically presented as mean ± SD (*n* = 4, ^**^ *p* ˂ 0.01 and ^***^ *p* ˂ 0.001, compared with the control group; ^##^ *p* ˂ 0.01 compared with H_2_O_2_ treatment group).

To select the optimal concentrations of fisetin to be used for the evaluation of the inhibitory efficacy of H_2_O_2_-induced cytotoxicity without causing cytotoxicity, ARPE-19 cells were cultured in a medium containing various different concentrations of fisetin for 24 h. The results of CCK8 assay revealed that cells treated with fisetin at 20 μM or less did not show a visible cytotoxic effect compared to untreated cells, whereas cell viability was significantly reduced at 30 μM or more (Figure 1A). To determine the suitable concentration of H_2_O_2_ for inducing oxidative damage, cells were stimulated to various amounts of H_2_O_2_ for the same period, and 500 μM, which reduced cell viability to approximately 60%, was selected and utilized in subsequent experiments (Figure 1B). To investigate the protective effect of fisetin on H_2_O_2_-induced cytotoxicity, cells were pretreated with the indicated concentrations of fisetin for 1 h and then exposed to H_2_O_2_ for 24 h. As shown in Figure 1C, the reduction in cell viability by H_2_O_2_ gradually improved with increasing fisetin pretreatment concentration.

### Fisetin Reduced H_2_O_2_-Induced Oxidative Stress in ARPE-19 Cells

**FIGURE 2:**
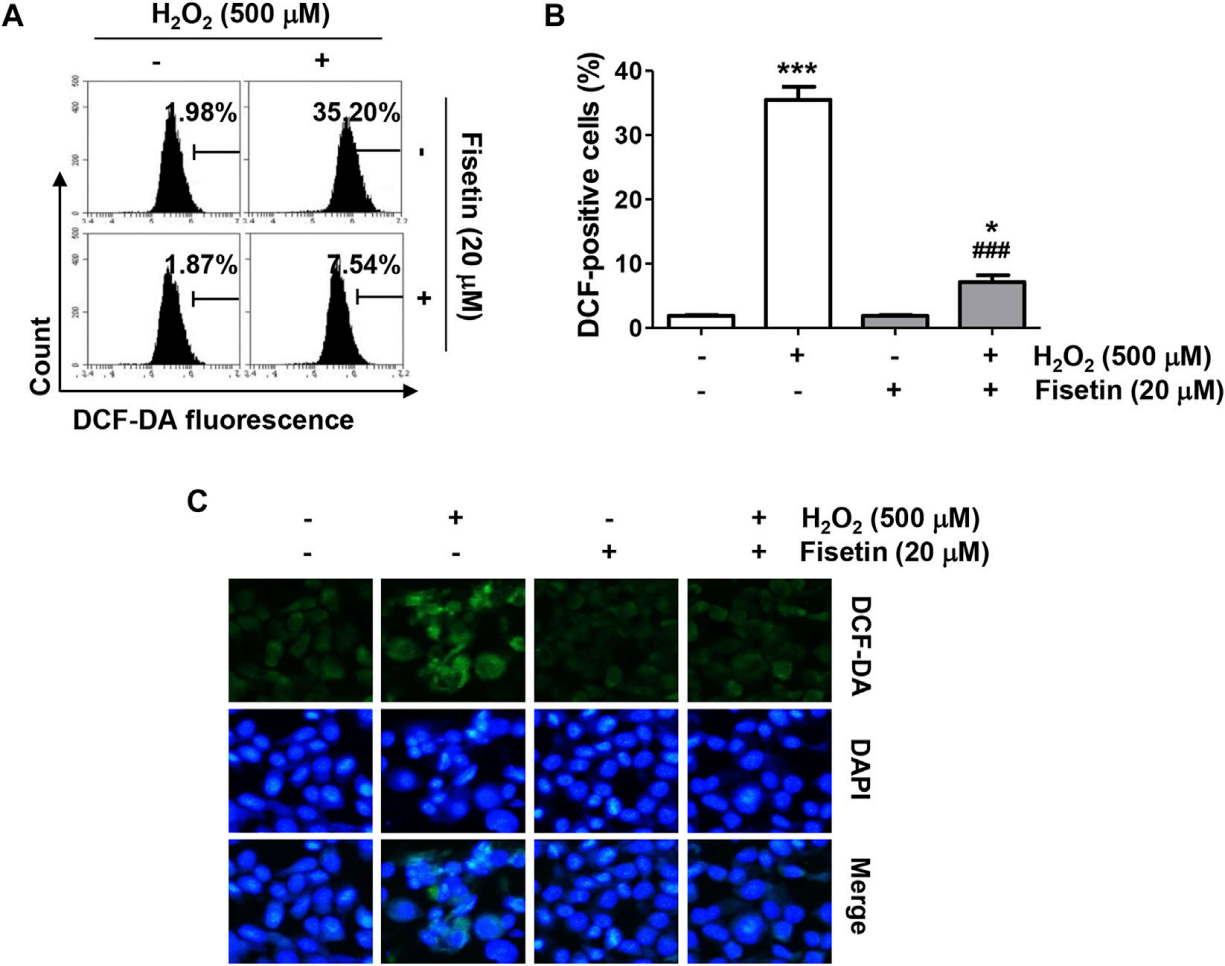
Fisetin attenuated H_2_O_2_-induced intracellular ROS generation in ARPE-19 cells. Cells were treated with or without 20 μM fisetin for 1 h and then treated with 500 μM H_2_O_2_ for 1 h. The levels of ROS production were determined using DCF-DA dye. **(A)** Representative results from flow cytometry analyses are shown. **(B)** The values of flow cytometry results are expressed as mean ± SD (*n* = 3, ^*^ *p* ˂ 0.05, and ^***^ *p* ˂ 0.001 compared with the control group; ^###^ *p* ˂ 0.001 compared with H_2_O_2_ treatment group). **(C)** The level of ROS (green) was observed under a fluorescence microscope, and representative images are presented. Nuclei were counterstained with DAPI (blue). Pretreatment of fisetin significantly suppressed increment of DCF-positive cell population following H_2_O_2_.

To evaluate the inhibitory efficacy of fisetin on H_2_O_2_-induced oxidative stress, the ROS scavenging ability of fisetin was evaluated. The results of flow cytometry data following DCF-DA staining showed that the level of ROS generation was markedly enhanced in H_2_O_2_-treated cells, which was significantly blunted by fisetin pretreatment (Figures 2A,B). The ability of fisetin to inhibit ROS generation was also evident in DCF-DA fluorescence images, the results of which are presented in Figure 2C. However, there was no significant difference in the level of ROS between cells treated with fisetin alone and control cells, confirming that fisetin has antioxidative property. These results indicate that the improved cell viability by fisetin in H_2_O_2_-treated ARPE-19 cells was related to its antioxidant effect.

### Fisetin Suppressed H_2_O_2_-Induced DNA Damage and Apoptosis in ARPE-19 Cells

**FIGURE 3:**
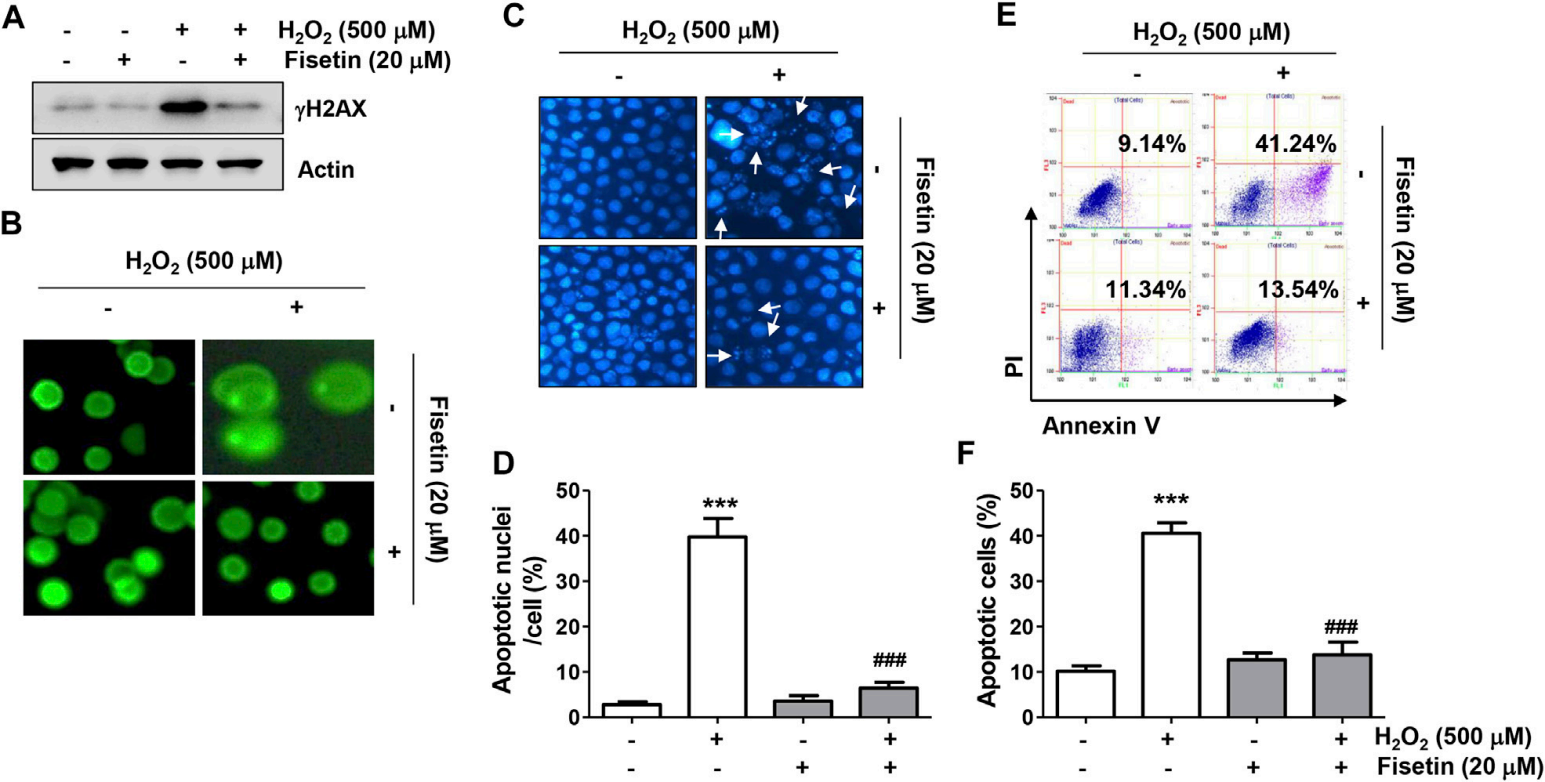
Fisetin protected ARPE-19 cells against H_2_O_2_-induced DNA damage and apoptosis. Cells were stimulated with or without 20 μM fisetin for 1 h and then treated with 500 μM H_2_O_2_ for another 24 h. **(A)** Levels of total p-γH2AX and actin were determined by immunoblotting using cell lysates. **(B)** Comet assay was performed to analyze DNA damage. Representative immunofluorescence microscopic images are shown. H_2_O_2_-induced increases in p-γH2AX expression and comet tail formation were apparently alleviated pretreatment of fisetin. **(C,D)** Cells were stained with DAPI for nuclear morphological observation under a fluorescence microscope for detection of apoptosis. **(C)** Representative images of DAPI-stained nuclei are shown. **(D)** Quantification results of nuclei with apoptotic cells are shown in a graph. H_2_O_2_-exposed cells displayed typical apoptotic properties such as nuclear shrinkage and fragmentation, whereas H_2_O_2_-mediated morphological changes were markedly restored by fisetin pretreatment. **(E,F)** After treatment, flow cytometry with annexin V/PI dual staining was performed. Representative histograms **(E)** and quantitative analysis **(F)** are shown. Pretreatment of fisetin significantly reduced the increased frequency of apoptotic cells following H_2_O_2_. **(D,F)** The graph represents the mean with SD (*n* = 3, ^***^ *p* ˂ 0.001, compared with the control group; ^###^ *p* ˂ 0.001 compared with H_2_O_2_ treatment group).

Next, we evaluated whether fisetin could protect ARPE-19 cells while suppressing oxidative stress-induced DNA damage and apoptosis. As illustrated in Figures 3A,B, the results of Western blot analysis and comet assay showed that the expression of the phosphorylated form of γH2AX (p-γH2AX), a sensitive biomarker for double-stranded DNA breaks, and the formation of DNA tails, indicating that single- and double-stranded DNA breaks have occurred, were increased in the cells treated with H_2_O_2_, indicating augmented DNA damage. However, in the presence of fisetin, H_2_O_2_-induced increases in p-γH2AX expression and comet tail formation were apparently alleviated. Moreover, H_2_O_2_-exposed cells displayed typical apoptotic properties such as nuclear shrinkage and fragmentation, as indicated by white arrows in Figures 3C,D, whereas intact nuclear morphology was maintained in untreated control cells and cells treated with fisetin alone. Consistent with these results, the flow cytometry results in Figures 3E,F demonstrated that a significant increase in the rate of apoptosis when cells were treated with H_2_O_2_ alone. However, addition of fisetin significantly reduced the increased frequency of apoptotic cells.

### Fisetin Mitigated H_2_O_2_-Induced Mitochondrial Dysfunction in ARPE-19 Cells

**FIGURE 4:**
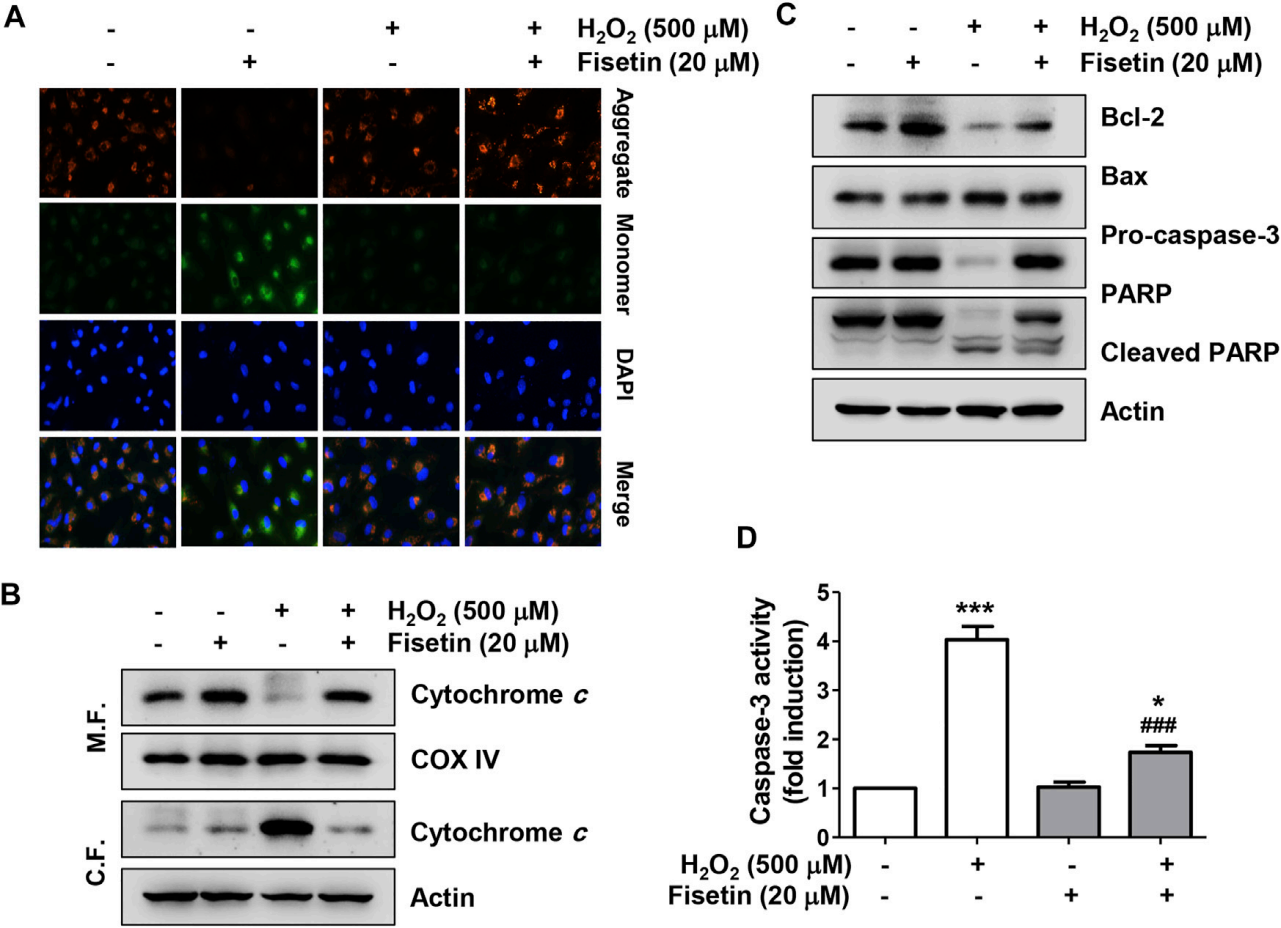
Fisetin ameliorated H_2_O_2_-induced mitochondrial dysfunction in ARPE-19 cells. Cells were treated with or without 20 μM fisetin for 1 h and then treated with 0.5 mM H_2_O_2_ for 24 h **(A)** mitochondrial membrane potential (MMP) was examined by the change in JC-1-derived fluorescence. Nuclei were counterstained with DAPI (blue). After staining, representative images of JC-1 aggregates (red, representing high potential) and monomers (green, representing low potential) of cells of each treatment group were captured using a fluorescence microscope. Pretreatment of fisetin reversed down-regulation of the ratio of JC-1 aggregates/monomers following H_2_O_2_-induced MMP depolarization. **(B)** To evaluate the expression and localization of cytochrome *c*, mitochondrial (M. F.) and cytoplasmic (C. F.) fractions were isolated from cells and assessed by Western blot analysis. cytochrome *c* was released from the mitochondria to the cytoplasm by H_2_O_2_, while pretreatment of fisetin was reversed H_2_O_2_-induced cytochrome c release. **(C)** Changes in the expression of Bcl-2, Bax, caspase-3, and PARP were confirmed using the extracted total proteins. **(D)** Caspase-3 activity was determined using a commercially available kit, and the concentration of p-nitroanilide released by activated caspase-3 was presented as a relative value compared to the control. H_2_O_2_ down-regulated the expression of anti-apoptotic Bcl-2 protein while up-regulating the expression of pro-apoptotic Bax, and induced the activation of caspase-3 and degradation of poly (ADP-ribose) polymerase (PARP). However, pretreatment of fisetin clearly reversed these H_2_O_2_-mediated alterations. All bar graphs represent mean ± SD (*n* = 3, ^*^ *p* ˂ 0.05, and ^***^ *p* ˂ 0.001 compared with control group; ^###^ *p* ˂ 0.01 compared with H_2_O_2_ treatment group).

We also investigated whether the anti-apoptotic effect of fisetin in H_2_O_2_-treated ARPE-19 cells was related to overcoming mitochondrial damage using the fluorescent probe 5,5,6,6′-tetrachloro-1,1′,3,3′ tetraethylbenzimi-dazoylcarbocyanine iodide (JC-1). As shown in Figure 4A, the intensity of red fluorescence, an aggregated form of the JC-1 complex, was strongly expressed in control cells, reflecting high levels of mitochondrial membrane potential (MMP) in the mitochondrial matrix of healthy cells. On the other hand, green fluorescence indicating that JC-1 remained in the monomeric form was highly increased in H_2_O_2_-treated cells compared with the control group, suggesting that MMP was depolarized. However, in cells pretreated with fisetin before being exposed to H_2_O_2_, the ratio of JC-1 aggregates/monomers remained at control levels, suggesting that H_2_O_2_-mediated mitochondrial dysfunction was effectively protected in the presence of fisetin. In addition, in the cells treated with H_2_O_2_, the expression of cytochrome *c* was strongly expressed in the cytoplasmic fraction, whereas it was lost in the mitochondrial fraction (Figure 4B), suggesting that cytochrome *c* was released from the mitochondria to the cytoplasm. Furthermore, H_2_O_2_ treatment significantly down-regulated the expression of anti-apoptotic Bcl-2 protein while up-regulating the expression of pro-apoptotic Bax, and induced the activation of caspase-3 and degradation of poly (ADP-ribose) polymerase (PARP) (Figures 4C,D). However, these changes were clearly reversed in the presence of fisetin.

### Fisetin Enhanced Nrf2-Mediated Activation of Heme Oxygenase-1 in H_2_O_2_-Treated ARPE-19 Cells

**FIGURE 5:**
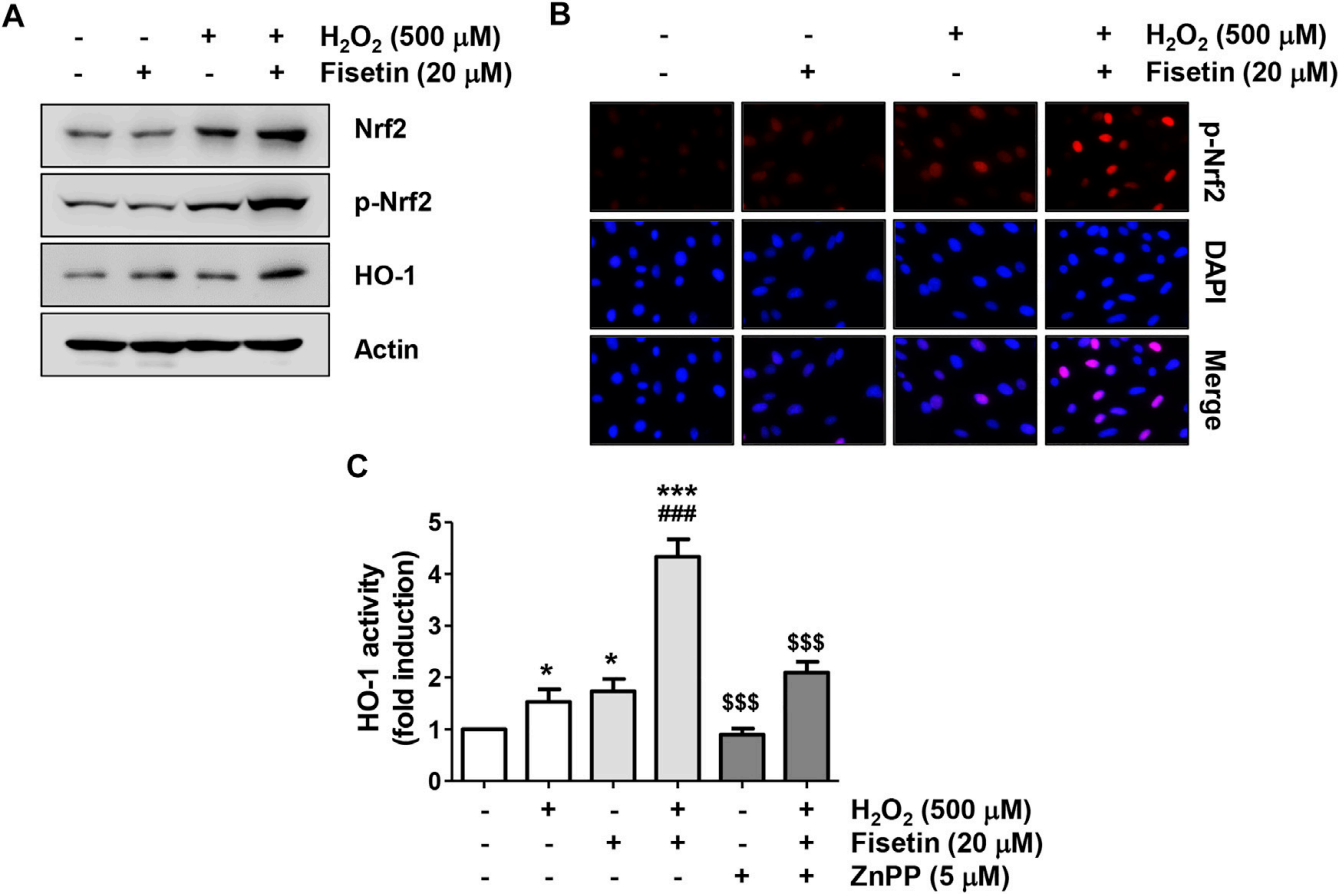
Fisetin activated Nrf2-mediated activation of HO-1 in H_2_O_2_-treated ARPE-19 cells. Cells were pretreated for 1 h with or without 20 μM fisetin or 5 μM ZnPP, and then treated with 500 μM H_2_O_2_ for another 24 h. **(A)** Total protein was isolated and followed by Western blotting was performed using antibodies against Nrf2, p-Nrf2, and HO-1. The expression of Nrf2 and HO1, as well as the phosphorylation of Nrf2 were enhanced by pretreatment of fisetin. **(B)** After treatment, cells were double-stained with p-Nrf2 (red) and DAPI (blue), and representative immunofluorescence images observed are shown. Phosphorylated form of Nrf2 was over-expressed in the nucleus following pretreatment of fisetin. **(C)** The activity of HO-1 was measured by the HO-1 activity assay kit. The increased activity of HO-1 in cells co-treated with fisetin and H_2_O_2_ was significantly restored in the presence of ZnPP, a specific inhibitor for HO-1. Results are presented as mean ± SD (*n* = 4, ^*^ *p* ˂ 0.05 and ^***^ *p* ˂ 0.001, compared with the control group; ^###^ *p* ˂ 0.001 compared with H_2_O_2_ treatment group. ^$$$^ *p* ˂ 0.001 compared with fisetin + H_2_O_2_ treatment group.

We further investigated whether the Nrf2 signaling was involved to clarify the mechanism by which fisetin mitigated oxidative stress in ARPE-19 cells. Western blot results in Figure 5A demonstrated that, the expression of Nrf2 as well as its phosphorylated form (p-Nrf2) was slightly increased in cells treated with H_2_O_2_ alone. However, but their expression was greatly upregulated in cells treated with H_2_O_2_ in the presence of fisetin (Figure 5A) and p-Nrf2 was predominantly expressed in the nucleus (Figure 5B), indicating that Nrf2 was translocated to the nucleus (Figure 5C). In addition, co-treatment of fisetin and H_2_O_2_ increased the expression and activity of HO-1, one of the downstream factors of Nrf2 (Figures 5A,C). However, the increased activity of HO-1 in cells co-treated with fisetin and H_2_O_2_ was significantly restored in the presence of ZnPP, a specific inhibitor for HO-1. These data suggest that fisetin might enhance the expression of HO-1 by promoting the transcriptional activity of Nrf2.

### Nrf2-Mediated Heme Oxygenase-1 Activation by Fisetin Was Required to Exert Antioxidant Properties Against H_2_O_2_ in ARPE-19 Cells

**FIGURE 6:**
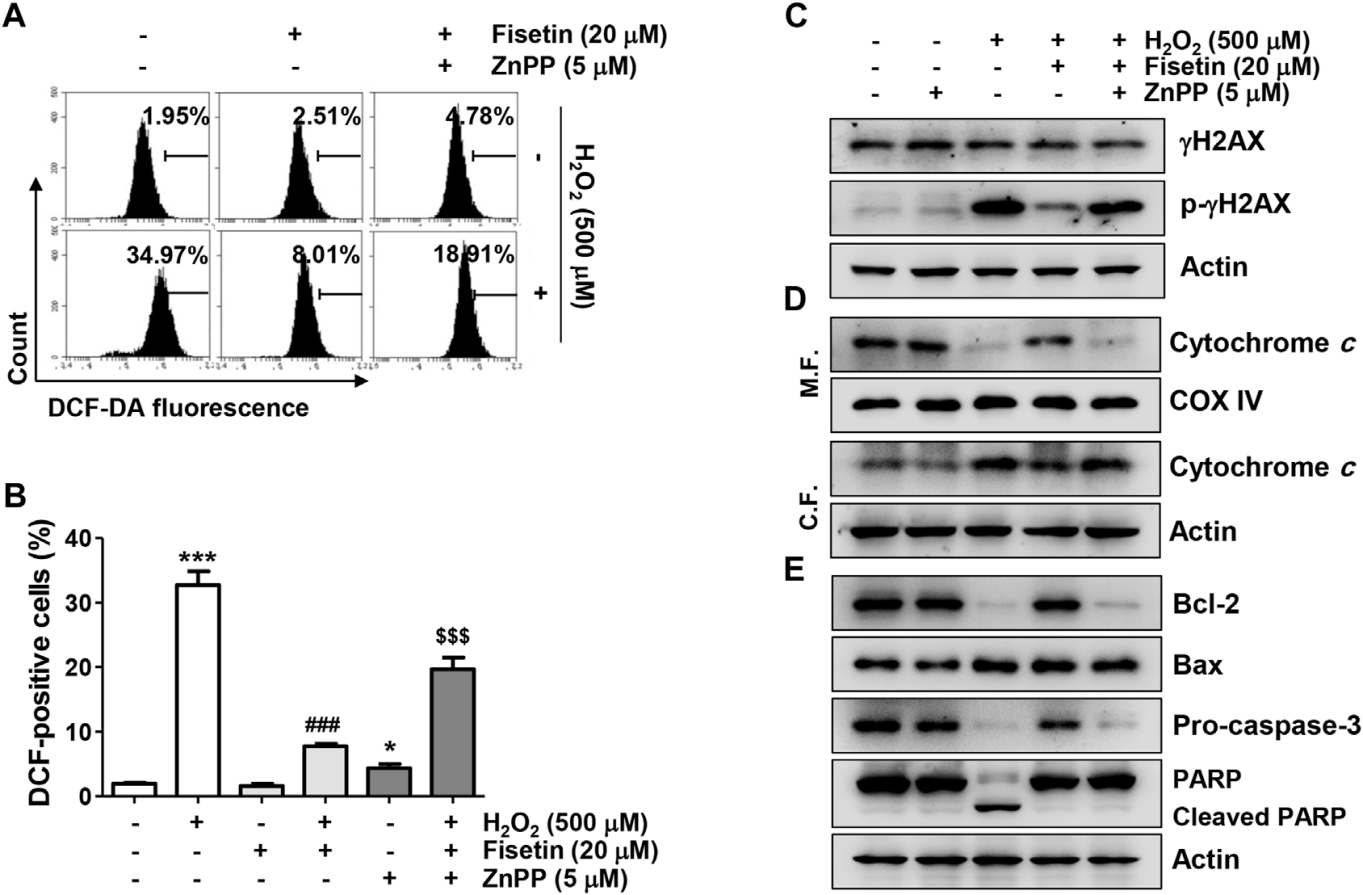
The antioxidative activity and the inhibitory effect on the expression of apoptosis-related factors of fisetin were disappeared in the presence of ZnPP in H_2_O_2_-treated ARPE-19 cells. Cells exposed to 20 μM fisetin and/or 5 μM ZnPP for 1 h were further treated with 500 μM H_2_O_2_ for 1 h **(A,B)** or 24 h **(C,D)**. **(A,B)** The levels of ROS generation were determined using DCF-DA dye. **(A)** Representative results from flow cytometry analyses are presented. **(B)** The graph represents the mean with SD (*n* = 4, ^*^ *p* ˂ 0.05 and ^***^ *p* ˂ 0.001 compared with control group; ^###^ *p* ˂ 0.001 compared with H_2_O_2_ treatment group; ^$$$^ *p* ˂ 0.001 compared with fisetin + H_2_O_2_ treatment group). Suppression of DCF-positive cells in the cells co-treated with fisetin and H_2_O_2_ was significantly restored in the presence of HO-1 inhibitor ZnPP. **(C–E)** The expression change of the indicated protein was confirmed using the extracted whole protein or mitochondrial and cytoplasmic proteins. The down-regulation of the expression of pro-apoptotic Bax, cleaved PARP, cytosolic cytochrome c, and p-γH2AX in the cells co-treated with fisetin and H_2_O_2_ was significantly restored by ZnPP.

**FIGURE 7:**
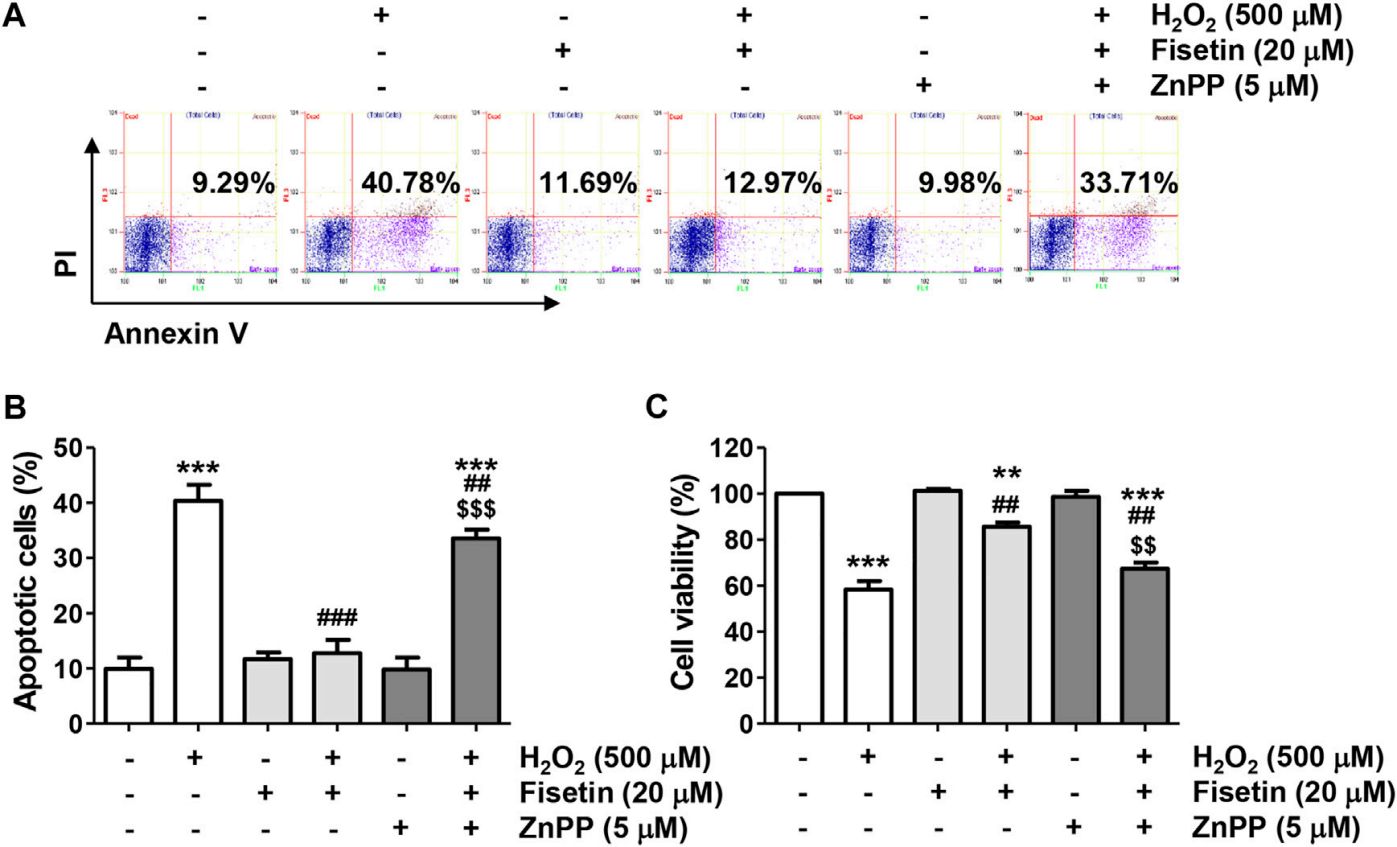
The blocking activity of fisetin on apoptosis and survival inhibitory effects was counteracted in H_2_O_2_-treated ARPE-19 cells. Cells treated with 20 μM fisetin and/or 5 μM ZnPP for 1 h were further treated with 500 μM H_2_O_2_ for 24 h **(A,B)** Flow cytometry with annexin V/PI dual staining was performed. Representative histograms **(A)** and quantitative analysis **(B)** are shown. **(C)** Cell viability was measured by the CCK8 analysis for cells cultured under the same conditions. The blocking effect of fisetin on H_2_O_2_-induced apoptosis was significantly counteracted by ZnPP, and the cytotoxic protective effect of fisetin was also significantly moderated. **(B,C)** Graphs represent means with SD (*n* = 3, ^**^ *p* ˂ 0.01, and ^***^ *p* ˂ 0.001 compared with control group; ^##^ *p* ˂ 0.01, and ^###^ *p* ˂ 0.001 compared with H_2_O_2_ treatment group; ^$$^ *p* ˂ 0.01 and ^$$$^ *p* ˂ 0.001 compared with fisetin + H_2_O_2_ treatment group.

We next evaluated the antioxidant potential of fisetin and its association with Nrf2-mediated HO-1 activation. As indicated in Figures 6A–C, the ROS scavenging activity and DNA damage blocking effect of fisetin in ARPE-19 cells exposed to H_2_O_2_ was significantly abolished in the presence of ZnPP. In addition, the inhibitory effects of fisetin on the release of cytochrome *c* into the cytoplasm and the expression change of apoptosis-related proteins, such as p-γH2AX, cytosolic cytochrome *c*, Bax, Bcle, caspase and cleaved PARP, in H_2_O_2_-treated cells were offset by inhibition of HO-1 activity (Figures 6D,E). Finally, we verified whether inhibition of HO-1ultimately result in offset of preventive effect of fisetin on H_2_O_2_-stimulated cellular damage. As shown in Figures 7A,B, blocking of HO-1 by ZnPP treatment markedly suppressed the preventive effect of fisetin on H_2_O_2_-induced apoptosis in ARPE-19 cells. Furthermore, the pre-treatment of ZnPP markedly decreased enhancing of cell viability by fisetin (Figure 7C). Therefore, these results showed that the blocking effect of fisetin on H_2_O_2_-induced apoptosis was significantly counteracted by ZnPP, and the cytotoxic protective effect of fisetin was also significantly moderated. Collectively, these data indicate that the protective effect of fisetin against H_2_O_2_-caused oxidative injury was at least related to Nrf2-mediated activation of HO-1 in ARPE-19 cells.

## Discussion

Recently, oxidative stress has been suggested as a critical factor in the pathogenesis of various retinal degenerative diseases (Chan et al., 2020; Dammak et al., 2021; Clementi et al., 2022). Therefore, the application of antioxidants to preserve RPE functional integrity from oxidative damage can be considered as one of the prospective approaches to reduce the pathogenesis of ocular diseases including retinal degeneration (Léveillard et al., 2019; Sadda et al., 2020). In this study, we provided evidence that fisetin blocked mitochondrial dysfunction, DNA damage and apoptosis by blocking H_2_O_2_-induced ROS generation in RPE ARPE-19 cells. In this study, activation of HO-1 following phosphorylation and nuclear translocation of the transcription factor Nrf2 was suggested as one of the mechanisms of the antioxidant action of fisetin, which reflects that fisetin acts as an activator of Nrf2 in RPE cells.

Fisetin, a naturally occurring flavonol, has been well demonstrated to possess a wide range of pharmacological potentials, including ROS scavenging activity, in a variety of *in vitro* and *in vivo* models. A few studies reported that fisetin inhibit epidermal growth factor-induced migration in ARPE-19 cells, and suppress cell death and inflammation on 4-hydroxynonenal mediated oxidative stress in ARPE-19 cells (Hytti et al., 2015; Lin et al., 2017). Nevertheless, the efficacy of fisetin in protecting RPE cells from oxidative stress is still not well understood. Therefore, the purpose of this study was to evaluate the ameliorating effect of fisetin on cellular damage based on its antioxidant activity using a well-established H_2_O_2_-treated oxidative stress-mimicking human RPE ARPE-19 cell model (Ung et al., 2017; Zhou et al., 2020b; Muangnoi et al., 2021). In this study, we reported that H_2_O_2_ treatment markedly decreased cell viability following induction of DNA damage, mitochondrial dysfunction, and apoptosis while increasing ROS levels in ARPE-19 cells. However, these pathological changes were counteracted by pretreatment of fisetin at concentrations in the non-cytotoxic range prior to H_2_O_2_-induced oxidative damage, confirming that the protective efficacy of fisetin was related to oxidative stress blockade.

Excessive accumulation of ROS in RPE cells due to disruption of the redox regulation signaling mechanisms and reduced free radical scavenging capacity can induce activation of RPE cell apoptosis pathways, contributing to the promotion of retinal degeneration processes (Park et al., 2019; Kaarniranta et al., 2020; Mahendra et al., 2020). Therefore, we investigated the ROS scavenging activity of fisetin and found that fisetin significantly repressed H_2_O_2_-induced ROS generation. This is in good agreement with the results of previous studies (Zhao et al., 2019; Rodius et al., 2020; Molagoda et al., 2021; Ding et al., 2022) and suggests that fisetin may act as an oxidative stress blocker. Several previous studies have proven that oxidative stress-induced apoptosis by H_2_O_2_ treatment in ARPE-19 cells is directly correlated with mitochondrial impairment (Zhao et al., 2020; Hong et al., 2021b; Kim et al., 2021b). Alteration of the outer mitochondrial membrane permeability by oxidative damage is linked to MMP dissipation. This ultimately results in the release of cytochrome *c* into the cytoplasm by inducing a loss of mitochondrial membrane integrity. In cytoplasm, cytochrome *c* enhances the activation of caspase cascade, triggering mitochondria-mediated apoptosis through degradation of substrate proteins of the activated effector caspases (Bock and Tait, 2020; Szabo et al., 2021). The regulation of this pathway, which is classified as an intrinsic apoptosis pathway, is highly dependent on the expression pattern of Bcl-2 family proteins to maintain mitochondrial homeostasis (Senichkin et al., 2020; Dadsena et al., 2021). In the present study, H_2_O_2_-treated ARPE-19 cells showed an increase in cytoplasmic expression of cytochrome *c* and a concomitant loss of MMP compared to untreated control cells. We also found that the relationship between Bax/Bcl-2 characterized by upregulated Bax and downregulated Bcl-2 protein levels was reversed in ARPE-19 cells exposed to H_2_O_2_. In addition, when ARPE-19 cells were exposed to H_2_O_2_, cleavage of PAPR and caspase-3 activity were greatly increased. However, all these changes caused by H_2_O_2_ were offset by the administration of fisetin, suggesting that fisetin rescued mitochondria-mediated apoptosis by preserving mitochondrial function in H_2_O_2_-treated ARPE-19 cells.

**FIGURE 8:**
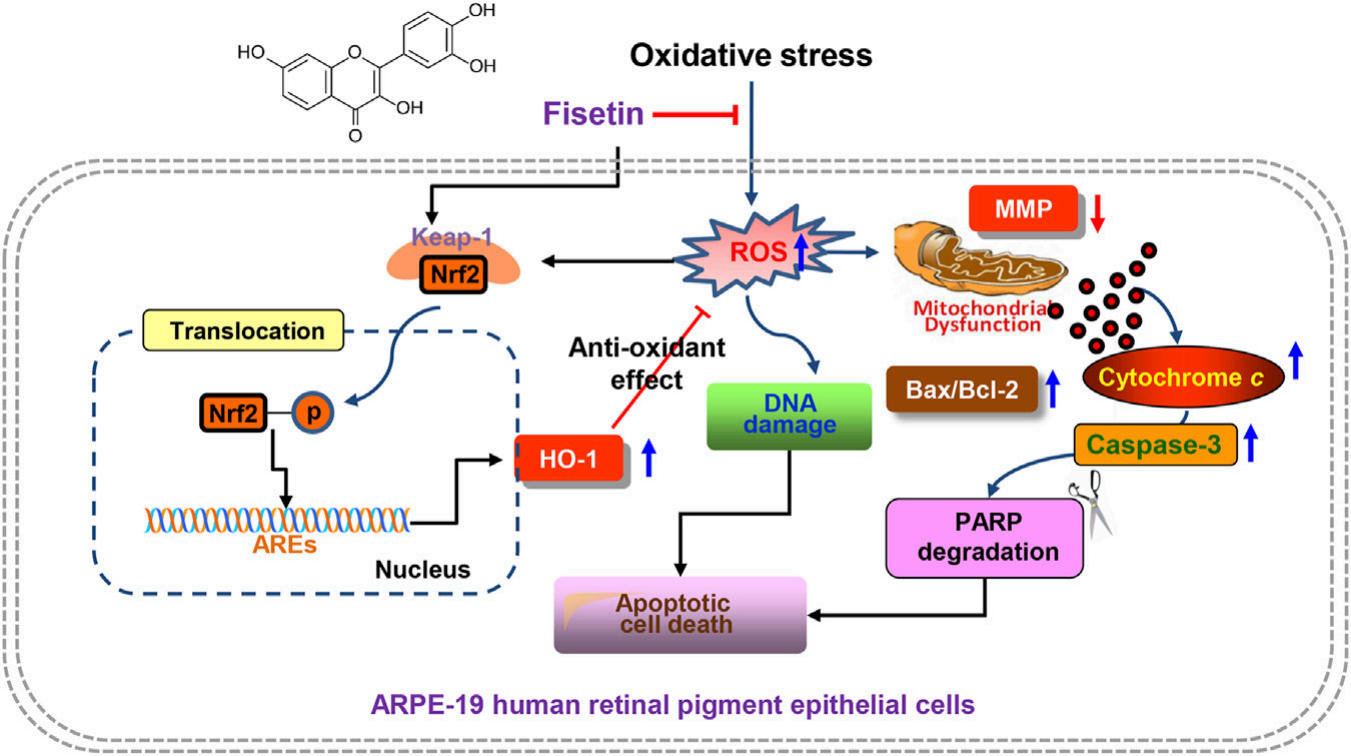
Graphical description of fisetin suppressing H_2_O_2_-induced oxidative damage in human RPE ARPE-19 cells through Nrf2-mediated HO-1 activation. In brief, fisetin significantly enhanced the expression of HO-1 along with activation of Nrf2 and reduced DNA damage, mitochondrial dysfunction and apoptosis in H_2_O_2_-treated ARPE-19 cells. However, these beneficial effects were abolished in the presence of an inhibitor of HO-1 activity, indicating that the enhanced expression of HO-1 contributed to the mitigating effect of fisetin on oxidative stress-induced cellular injury. Taking together, these results provide strong evidence that fisetin as a ROS scavenger can rescue RPE cells from oxidative damage through activation of the Nrf2/HO-1 axis.

Certain intracellular signaling pathways are involved in defense strategies against oxidative stress, and there is growing evidence that the Nrf2/AREs pathway is involved in the antioxidant activity of fisetin in multiple cell lines (Sim et al., 2020; Jiang et al., 2021; Li et al., 2022). There is growing evidence that Nrf2 is an effective target in the regulation of oxidative stress-related retinal degeneration. For example, it has been reported that negative regulation of Nrf2 is implicated in the pathogenesis of maculopathy (Datta et al., 2017; Bellezza, 2018; Hyttinen et al., 2019), and mice deficient in Nrf2 have been shown to exhibit retinal alterations similar to AMD (Zhao et al., 2014; Rowan et al., 2020). On the other hand, Nrf2-mediated activation of HO-1 was effective in ameliorating oxidative damage in human RPE cells (Lambros and Plafker, 2016; Hyttinen et al., 2019; Hong et al., 2021b). In the meantime, many previous results have demonstrated that activation of HO-1 by some phytochemicals with antioxidant activity plays an important role in the resistance of ARPE-19 cells to oxidative stress (Zhao et al., 2020; Chen et al., 2021; Zhang et al., 2022). As is well known, Nrf2 is normally sequestered in the cytoplasm by binding to its inhibitor Kelch-like ECH-associated protein 1 (Keap1). However, when cells are exposed to oxidative stimulation or in the presence of Nrf2 activators, Nrf2 is dissociated from Keap1. For the transcriptional activity of ARE-controlled phase II detoxification enzymes and other antioxidant enzymes including HO-1, Nrf2 must be phosphorylated before translocation to the nucleus (Tavakkoli et al., 2019; Ulasov et al., 2022). Our results showed that fisetin remarkably enhanced the expression and phosphorylation of Nrf2 as well as its expression in the nucleus in H_2_O_2_-treated ARPE-19 cells. Fisetin also improved the expression and enzymatic activity of HO-1, suggesting that fisetin was able to activate the Nrf2/AREs pathway, which was in good agreement with previous findings (Hanneken et al., 2006; Sim et al., 2020; Jiang et al., 2021; Li et al., 2022). However, the ROS scavenging activity and viability-enhancing ability of fisetin were significantly abolished by blocking HO-1 activity via a HO-1 antagonist ZnPP. Moreover, fisetin-mediated alleviation of H_2_O_2_-induced DNA damage and apoptosis was reversed in the presence of ZnPP. Taken together, the activation of Nrf2/HO-1 signaling could be closely related to maintaining mitochondrial integrity and redox homeostasis under oxidative conditions (Figure 8). However, further studies, including the role of upstream intracellular signaling pathways involved in the phosphorylation of Nrf2 by fisetin, and its efficacy in primary human RPE cells and *in vivo* animal models, need to be performed. Furthermore, further studies are warranted to identify whether this protective effect of fisetin against oxidative stress on other source of RPE cells and other ocular cells, such as corneal epithelial cells and conjunctival epithelial cells.

In summary, here we demonstrated that fisetin significantly enhanced the expression of HO-1 along with activation of Nrf2 and reduced ROS generation, DNA damage, mitochondrial dysfunction and apoptosis in H_2_O_2_-treated ARPE-19 cells. However, these beneficial effects were abolished in the presence of an inhibitor of HO-1 activity, indicating that the enhanced expression of HO-1 contributed to the mitigating effect of fisetin on oxidative stress-induced apoptotic cellular injury. Taking together, these results provide strong evidence that fisetin as a ROS scavenger can rescue RPE cells from oxidative damage through activation of the Nrf2/HO-1 axis. Although further studies still remain to be addressed, our results suggested that fisetin may be a potential therapeutic strategy for the prevention and treatment of retinal disorders related to oxidative stress.

## Data Availability

The original contributions presented in the study are included in the article/supplementary materials, further inquiries can be directed to the corresponding authors.
